# Headspace Solid-Phase Microextraction Analysis of Volatile Components in Peanut Oil

**DOI:** 10.3390/molecules26113306

**Published:** 2021-05-31

**Authors:** Kai-Min Yang, Louis Kuoping Chao, Chin-Sheng Wu, Zih-Sian Ye, Hsin-Chun Chen

**Affiliations:** 1 Department of Hospitality Management, Mingdao University, Changhua 523, Taiwan; a9241128@gmail.com; 2 Department of Cosmeceutics, China Medical University, Taichung 406, Taiwan; kuoping@mail.cmu.edu.tw (L.K.C.); cswu@mail.cmu.edu.tw (C.-S.W.)

**Keywords:** peanut oil, *Arachis hypogaea*, HS-SPME, GC, pyrazines

## Abstract

Peanut oil is favored by consumers due to its rich nutritional value and unique flavor. This study used headspace solid-phase microextraction (HS-SPME) combined with gas chromatography (GC) and gas chromatography–mass spectrometry (GC-MS) to examine the differences in the peanut oil aroma on the basis of variety, roasting temperatures, and pressing components. The results revealed that the optimal conditions for extracting peanut oil were achieved through the use of 50/30 μm DVB/CAR/PDMS fibers at 60 °C for 50 min. The primary compounds present in peanut oil were pyrazines. When peanuts were roasted, the temperature raised from 120 °C to 140 °C and the content of aldehydes in peanut oil increased; however, the content of aldehydes in No. 9 oil at 160 °C decreased. The components of peanut shell oil varied depending on the peanut variety. The most marked difference was observed in terms of the main compound at the two roasting temperatures. This compound was a pyrazine, and the content increased with the roasting temperature in hekei oils. When the roasting temperature was lower, No. 9 oil contained more fatty acid oxidation products such as hexanal, heptanal, and nonanal. When the roasting temperature increased, No. 9 oil contained more furfural and 5-methylfurfural. Heren oil was easier to oxidize and produced nonanal that possessed a fatty aroma.

## 1. Introduction

Peanut is an annual herb of the Leguminosae or Fabaceae family and genus *Arachis* that is native to South America, Mexico, and Central America. It is widely distributed in tropical and temperate regions [1,2]. It is rich in fatty acids that are primarily comprised of unsaturated fatty acids such as oleic acid (40–56%) and linoleic acid (16%) [3]. Peanuts are one of the main oil crops produced throughout the world [4], and they possess a variety of biologically active compounds that make them an important source of economic crops and plant proteins [5].

Roasting of oil prior to pressing has been demonstrated to be an effective means of improving the flavor of aromatic oils. This is due to the presence of many complex reactions that occur during roasting, including the Maillard reaction, Strecker degradation, lipid oxidation, and caramelization, which all serve to produce a large number of volatile compounds [6,7] that affect the flavor of oil products. The use of hot pressing increases the oil yield of peanuts and produces a nutty aroma. Peanuts exhibit a strong nutty flavor under heat treatment at 100–150 °C [8]. Hot-pressed peanut oil is subjected to material selection, roasting, and physical pressing that is facilitated by natural fiber filtration [9], and the method for squeezing peanut oil includes taking roasted peanuts that have been crushed and steamed before filling them into round cake molds that are subsequently arranged and stacked. The spilled grease is then squeezed with an external force [10]. The volatile compounds in oil are key indicators that determine the organoleptic properties of vegetable oils [11]. Aromatic roasted peanut oil is a traditional Chinese edible oil [12] that possesses a unique strong nutty taste that distinguishes it from different edible vegetable oils [11], and the Maillard reaction and lipid oxidation are very important for flavor formation. Most volatile compounds with odor activity are lipid derivatives and intermediate products of the Maillard reaction [6].

The solid-phase microextraction method (SPME) has been steadily growing since 1990 [13]. It is the most used technique for the extraction of volatile compounds because of its easy, fast, and solvent-free operation [14]. Dun et al. [15] used HS-SPME combined with GC–MS–O to identify volatile compounds in hot-pressed peanut oil; Liu et al. [11] used HS-SPME combined with GC–MS to observe the changes in volatile composition of the roasted peanut oil during the roasting process.

According to the literature regarding different processing conditions for other nuts such as pistachios, the nut variety, pressing position, and baking temperature all affect the volatile components of the oil [16]. Peanut oil produces an aroma when heated. Although numerous studies exist examining the aroma of peanut oil, there are only a few studies regarding the effect of processing conditions on the aroma of peanut oil. Therefore, this study used HS-SPME combined with GC and GC–MS to explore the differences in the volatile components of peanut oil using different roasting temperatures, peanut varieties, and pressing conditions. The results of this study can serve as a reference for related agriculture and oil processing fields.

## 2. Results and Discussion

### 2.1. Optimal Absorption Fiber

**Figure 1:**
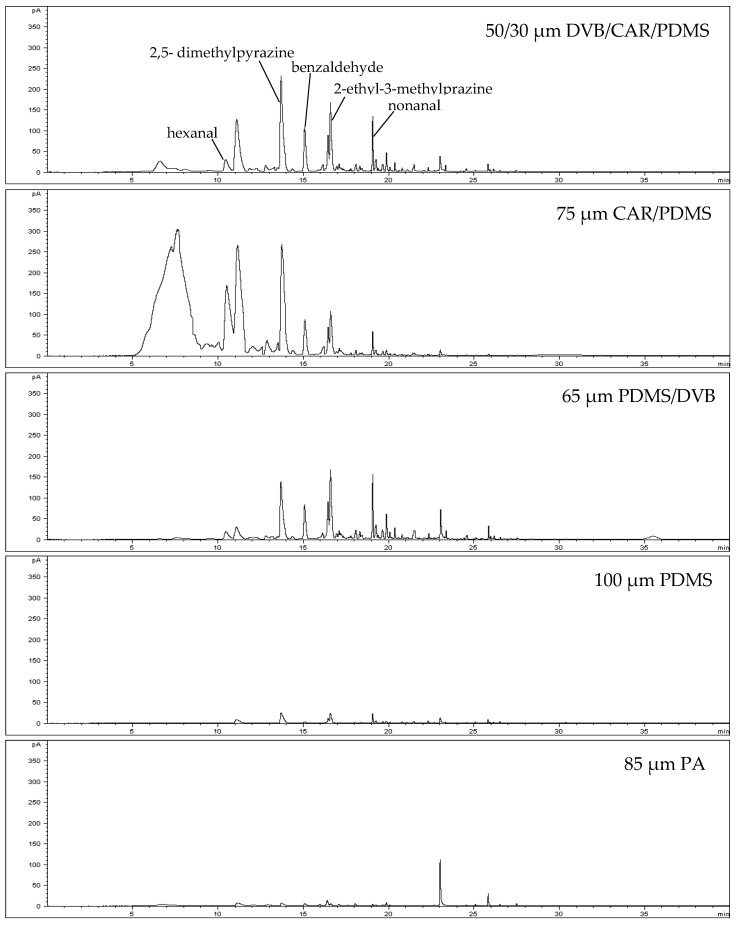
Gas chromatograms of Hekei-140 volatile components using different absorption fibers in combination with HS-SPME.

In this study, five different adsorption fibers were used, including 50/30 μm divinylbenzene/carboxen/polydimethylsiloxane (DVB/CAR/PDMS), 65 μm polydimethylsiloxane/divinylbenzene (PDMS/DVB), 75 μm polydimethylsiloxane/carboxen (CAR/PDMS), 85 μm polyacrylate (PA), and 100 μm polydimethylsiloxane (PDMS), to extract volatile components in peanut oil. For black shell-140, the results indicate that 75 μm CAR/PDMS exhibited poor separation efficiency for compounds at 10 min prior to the retention time, and 65 μm PDMS/DVB exhibited poor adsorption capacity for small molecules. Therefore, the use of 50/30 μm DVB/CAR/PDMS produced a better effect on peanut oil and exhibited excellent extraction capacity (Figure 1).

The polarity of a fiber is an important criterion for extraction. Different fiber coatings affect the types of adsorbed volatile compounds. Compared to DVB/CAR/PDMS, CAR/PDMS fibers are more suitable for adsorbing low-molecular-weight compounds; however, as the DVB/CAR/PDMS fiber is bipolar, both polar and nonpolar compounds can be extracted. Because DVB, CAR, and PDMS are highly sensitive to polar, small-molecule, and nonpolar volatile compounds, respectively, using a 50/30 μm DVB/CAR/PDMS adsorption fiber can extract a greater amount of volatile components from edible oil.

The 50/30 μm DVB/CAR/PDMS fiber is typically used for flavor analysis; it is particularly effective for pyrazine extraction [17] and is most suitable for identifying volatile oxygen-containing substances in vegetable oils. This method has been successfully used to track oil and the formation of volatile substances (such as hexanal, *trans*-2-hexenal, *trans*-2-heptenal, nonanal, 2-pentylfuran, and 1-octen-3-ol) when the product is oxidized [18].

### 2.2. Optimum Adsorption Temperature

**Figure 2:**
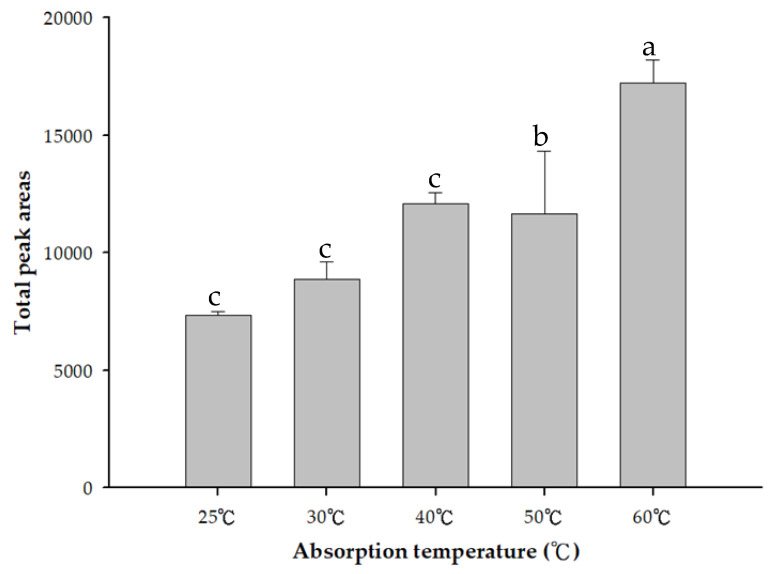
Comparison of the contents of the total volatiles in Hekei-140 at different absorption temperatures using HS-SPME. The data correspond to the mean ± SD of triplicate experiments. ^a–c^: Values possessing different superscripts are significantly different (*p* < 0.05).

In the HS-SPME adsorption fiber test, this study used 50/30 μm DVB/CAR/PDMS adsorption fibers with better adsorption capacity to adsorb peanut oil at 25, 30, 40, 50, and 60 °C. For analysis of the total black shell-140 content of volatile components, the results demonstrate that the total volatile components of Hekei-140 adsorbed at 60 °C were the highest (Figure 2) Therefore, 60 °C was selected for the subsequent experiments.

The extraction temperature exerted a significant effect on large-molecular-weight compounds such as tetradecanol and hexadecanol. A higher extraction temperature resulted in a larger peak area; however, the effect on the more volatile compounds (such as 3-methylbutanal) was less obvious [19]. A higher temperature provides sufficient energy for the volatile compounds to increase the vapor pressure, and this is conducive to the release of volatile compounds into the headspace. Additionally, the high temperature can drive the tendency to move volatile substances from the sample to the fiber coating.

Peanuts are rich in unsaturated fatty acids. The ability of unsaturated fatty acids to resist free-radical attacks is weaker than that of saturated fatty acids. However, even saturated fatty acids are oxidized at temperatures above 60 °C. These oxidation products contain volatile compounds [20,21]. According to the literature, the maximum temperature for this experiment was set to 60 °C.

### 2.3. Optimal Adsorption Time

**Figure 3:**
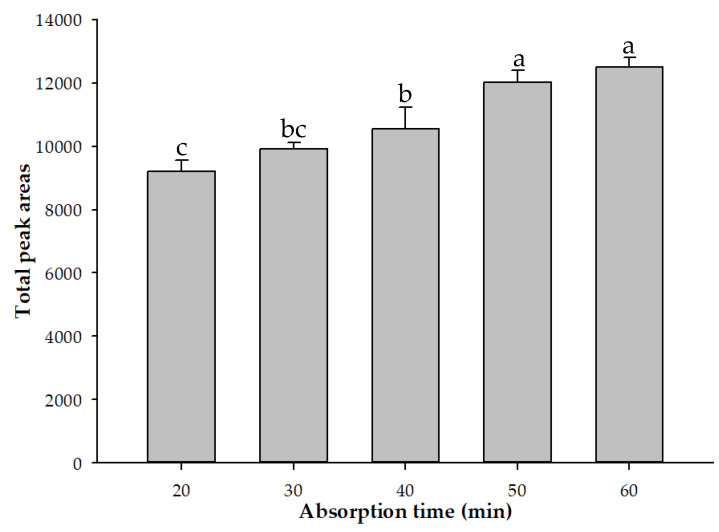
Comparison of the contents of the total volatiles in Hekei-140 at different absorption times using HS-SPME. The data correspond to the mean ± SD of triplicate experiments. ^a–c^: Values possessing different superscripts are significantly different (*p* < 0.05).

For the HS-SPME adsorption fiber and adsorption temperature test, a 50/30 μm DVB/CAR/PDMS adsorption fiber possessing a better adsorption capacity was used in this study. The test was performed at 10, 20, 30, 40, 50, and 60 min at a water bath temperature of 60 °C. When examining the total volatile components of Heijingang shelled peanuts at adsorption times up to 60 min, the results indicated that the total volatile component content of Heijingang shelled peanuts was the highest when the adsorption time was 50 min. The total volatile content of the peanuts did not increase when the adsorption time was increased to 60 min (Figure 3). Therefore, 50 min was chosen for the subsequent experiments.

When the adsorption extraction time was increased to more than 45 min, the peak area of the volatile components decreased significantly. This is due to observation that the adsorption time affected the extraction efficiency. Extending the adsorption time may affect the sensitivity of the fiber. This phenomenon depends on the affinity of the compound for the fiber. The optimal adsorption time for each compound was due to the competitive absorption of the compound on the fiber coating. Differences have previously been observed [22]; in the HS-SPME analysis process, as the extraction time increases, the analyte diffuses back from the fiber into the headspace and reaches a dynamic equilibrium, ultimately resulting in a decrease in the extraction concentration [23].

### 2.4. Differences in Volatile Components of Peanut oil Prepared at Different Roasting Temperatures

The composition of volatiles in the oil samples was different, and a total of 66 volatile components were identified (Table 1). The results show that the primary components of peanut oil at the roasting temperature of 120 °C were hexanal, nonanal, methyl pyrrol-2-yl ketone, *p*-ethylphenol, 2-methylpyrazine, 2,5-dimethylpyrazine, 2-ethyl-6-methylpyrazine, 2-ethyl-3-methylpyrazine, and 2-ethyl-3,6-dimethylpyrazine. When the roasting temperature was 140 °C, the primary components of peanut oil were hexanal, dodecane, undecane, methyl pyrrol-2-yl ketone, 2-methoxy-4-vinylphenol, 2, 5-dimethylpyrazine, 2-dimethylpyrazine, and 2-ethyl-3,6-dimethylpyrazine. The primary components of peanut oil at the roasting temperature of 160 °C were (*E*)-2-heptenal, nonanal, *p*-vinylphenol, 2-methylpyrazine, 2,5-dimethylpyrazine, 2-ethyl-6-methylpyrazine, 2-ethyl-3-methylpyrazine, and 2-ethyl-3,5-dimethylpyrazine.

The aldehydes produced by the oxidation of fatty acids included hexanal, nonanal, (*E*)-2-heptenal, 2-octenal, (*E*)-2-nonenal, (*E*)-2-decenal, (2*E*,4*Z*)-decadienal, (2*E*,4*E*)-decadienal, and benzaldehyde [24]. According to previous studies, the pyrazines present in peanut oil are the source of roasting flavor [25], and pyrazines are the products of the Mener reaction that occur in meat, fish, cocoa, nuts, popcorn, and bread [26].

**Figure 4:**
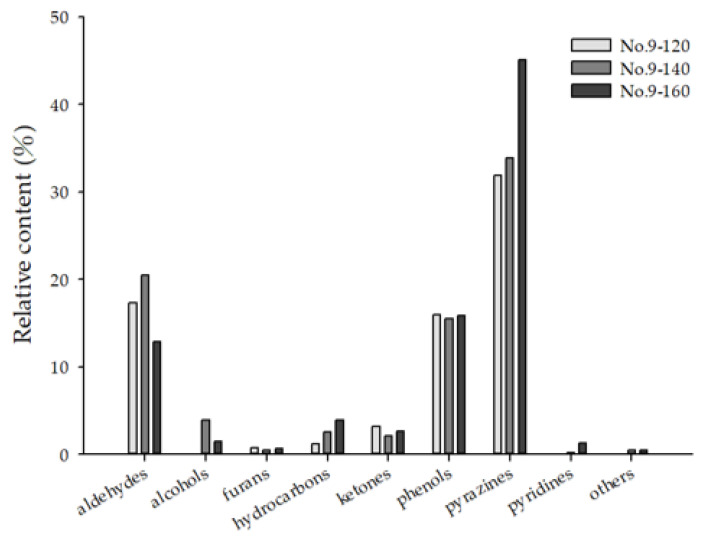
Classification of volatile compounds in Tainan S. No. 9 peanut oils using HS-SPME.

From the data presented in Figure 4, it can be observed that the primary components of Tainan S. No. 9 peanut oil were pyrazines, and their content was increased greatly at high temperatures (160 °C). Such compounds are the products of the Maillard reaction, which increases their content upon an increase in the processing temperature. Two compounds related to the roasting taste were produced, including 2,5-dimethylpyrazine and 2-ethyl-3,5-dimethylpyrazine. These pyrazines are considered to be the key factors that cause peanut oil to possess a strong nutty flavor [11]. The content of aldehydes in Tainan S. No. 9 peanut oil decreased when the roasting temperature increased to 160 °C. Previous studies have suggested that this may be due to some aldehydes being highly volatile compounds that may be lost due to evaporation during heating. 

**Figure 5:**
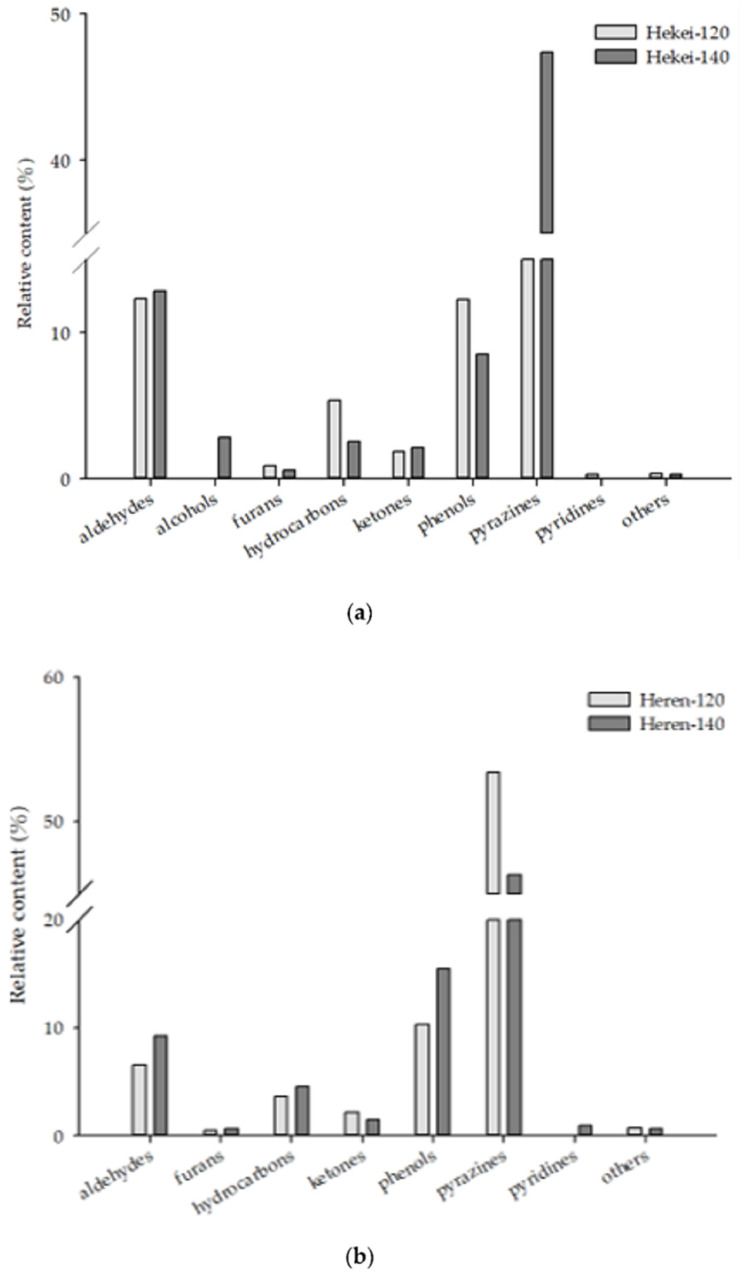
Classification of volatile compounds in Tainan S. No. 16 peanut oils using HS-SPME. (**a**) Tainan S. No. 16 with peanut shell peanut oil at different roasting temperatures. (**b**) Tainan S. No. 16 peanut oil at different roasting temperatures.

Additionally, benzene within the sample changes the content of cyclic derivatives, and alcohols may be related to water loss and the roasting process [27]. In hekei oil, the content of aldehydes and pyrazines increased as the roasting temperature increased (Figure 5a). The former was caused by the formation of aldehydes by the degradation of amino acids through Strecker degradation [13,27], and the latter was the product of the Maillard reaction, which increased their content due to the increase in processing temperature [13]. 

In heren oil, the content of aldehydes increased as the roasting temperature increased. The reason for this increase was the same as that described above; however, the content of pyrazines decreased in response to an increase in roasting temperature (Figure 5b), with the exception of 3,6-dimethylpyrazine which increased (Table 1). It has been reported that primary pyrazines change from 2,5-dimethylpyrazine and trimethylpyrazine to 2,5-dimethylpyrazine, 2,6-dimethylpyrazine, trimethylpyrazine, and 2-ethyl-3,6-dimethylpyrazine [28].

### 2.5. Differences in Volatile Components of Peanut Oil Made from Different Varieties

Eight types of aldehydes, three types of alcohols, one type of furan, six types of hydrocarbons, two types of ketones, five types of phenols, 15 types of pyrazines, one type of pyridine, and two types of hekei oils roasted at 120 °C and 140 °C were identified. Other compounds, including 12 types of aldehydes, one type of alcohol, two types of furans, six types of hydrocarbons, three types of ketones, five types of phenols, 14 types of pyrazines, and one unknown compound, were identified in the No. 9 oil of Tainan selected roasted at 120 °C and 140 °C. The types of pyridines and the other compound are listed in Table 1.

**Figure 6:**
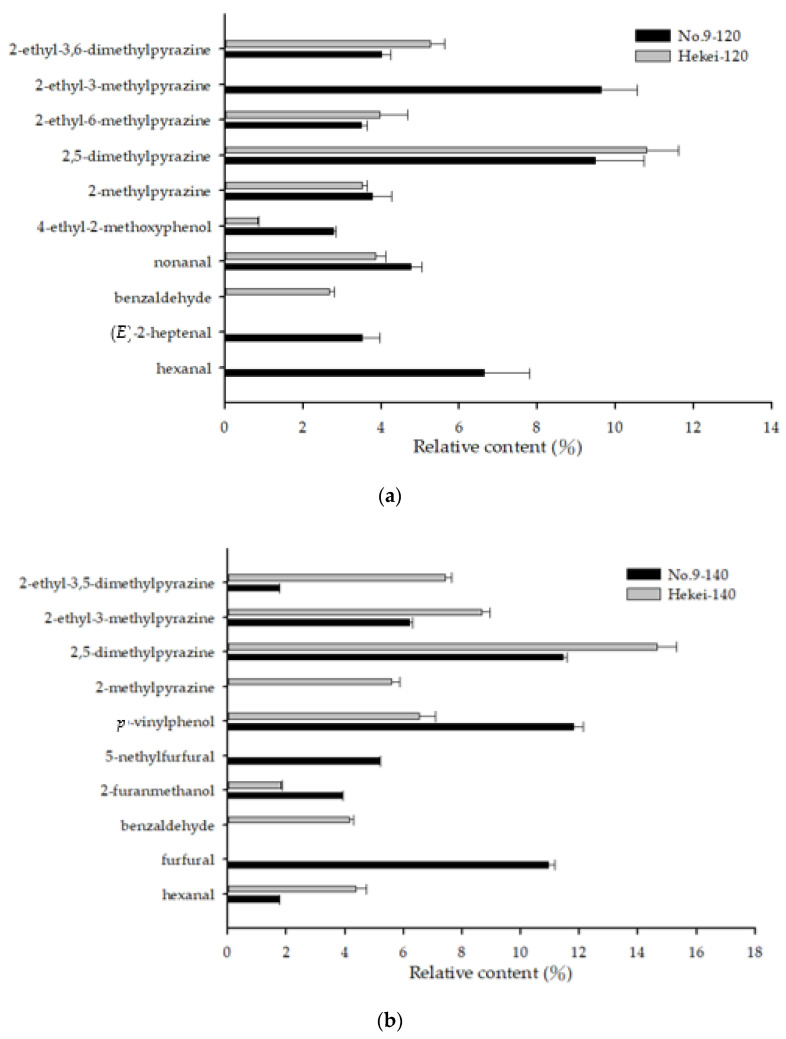
The main volatile compounds of different varieties of peanut oil: (**a**) peanut oil roasted at 120 °C; (**b**) peanut oil roasted at 140 °C.

The primary components of the two varieties at different roasting temperatures are shown in Figure 6. At 120 °C, the hekei oil was rich in 2-ethyl-3,6-dimethylpyrazine and 2-ethyl-6-methylpyrazine, while 5-dimethylpyrazine, 2-ethyl-3-methylpyrazine, hexanal, and (*E*)-2-heptenal were only identified in No. 9 oil. At 140 °C, hekei oil contained greater amounts of 2-ethyl-3,5-dimethylpyrazine, 2,5-dimethylpyrazine, 2-ethyl-3-methylpyrazine, and hexanal, while benzaldehyde was only identified in this sample. The primary components of the No. 9 oil selected in Tainan were *p*-vinylphenol, 5-methylfurfural, and furfural.

When the roasting temperature was low, the No. 9 oil contained more aldehydes. Among them, hexanal, heptanal, and nonanal are the products of fatty-acid oxidation and can produce peculiar smells that affect the oil flavor [29]. In a study by Toschi et al. [30], it was determined that the fatty-acid composition of different varieties of cashew nuts is different, and the oxidation of the oil is affected by the fatty-acid composition of the oil and the processing methodology, while the concentrations of oxygen and free fatty acids are affected by factors such as the interaction of these factors on the oxidation of oil [31]. Therefore, this phenomenon may have been caused by the different fatty-acid compositions and processing temperatures of Tainan S. No. 9 and Tainan S. No. 16 peanuts.

At higher roasting temperatures of No. 9 oil, the Maillard reaction induced the formation of furfural and 5-methylfurfural [32]. It is speculated that these two compounds do not react with amino acids. The reaction resulted in a lower content of pyrazines in this sample, and no furfural was identified in the hekei oil. It may be that, in this sample, furfural underwent downstream reactions, and the difference in the reaction path may have been due to the different amino-acid contents present in the different varieties.

### 2.6. The Differences in Volatile Components of Peanut Oil in Different Pressing Parts

**Figure 7:**
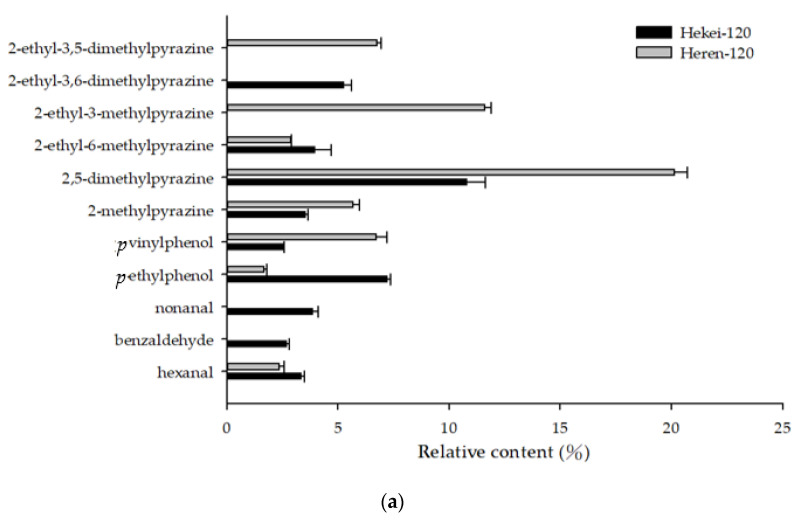

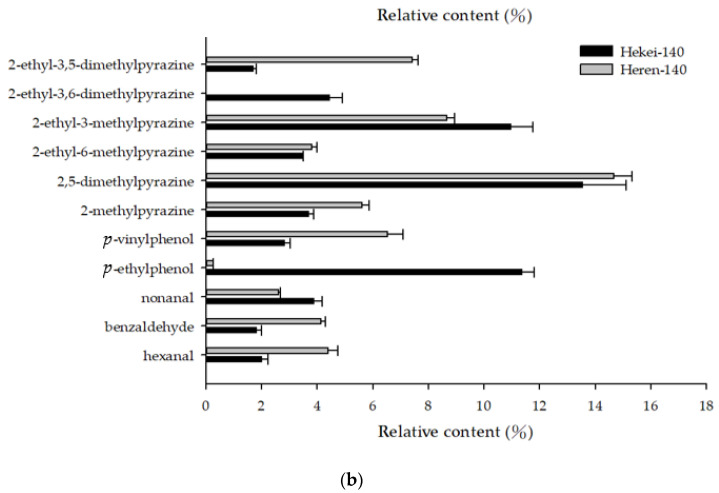
The main volatile compounds of different parts of peanut oil: (**a**) peanut oil roasted at 120 °C; (**b**) peanut oil roasted at 140 °C.

The contents of aldehydes, alcohols, and hydrocarbons in hekei oil were more abundant than those in heren oil (Table 1). When the roasting temperature was 120 °C, the primary component in both shell oils was 2,5-dimethylpyrazine (12.95–14.65%). The content of pyrazines in heren oil was higher than was that in hekei oil. Hekei oil contained more linear aldehydes formed by the oxidation of fatty acids such as hexanal and nonanal. The contents of phenols in the primary components of the two oils were not significantly different. Increasing the temperature increased the content of nonanal and *p*-ethylpheol in heren oil, decreased the content of 2,5-dimethylpyrazine and 2-ethyl-3,5-dimethylpyrazine, and increased the content and types of pyrazines in hekei oil, among which 2-ethyl-3,5-dimethylpyrazine (increased to 7.41% without detection), 2-ethyl-3-methylpyrazine (increased to 8.66% without detection), and 2,5-dimethylpyrazine (increased from 10.80 to 14.65%) were increased significantly. At higher processing temperatures, hekei oil produces more pyrazines, while heren oil was easier to oxidize and produced nonanal (Figure 7).

Ojeda-amador et al. [13] found that, in addition to more hexanal and nonanal, pistachio oil that is pressed in the shell also contains more alcohols, hydrocarbons, terpenes, and aromatic volatile compounds. It is likely that the shell can generate greater friction during pressing, ultimately promoting the extraction of volatiles.

## 3. Materials and Methods

### 3.1. Oil Material

The samples were obtained from Tainan S. No. 9 and Tainan S. No. 16 peanuts harvested in the winter of 2018 (Chiayi, Taiwan). They were commissioned to be produced by the Lao Gongyi Oil Company (Changhua, Taiwan). The roasting temperatures used were 120 °C, 140 °C, and 160 °C. Tainan S. No. 9 peanut oil was pressed in the shell, and Tainan S. No. 16 peanut oil was divided into shelled and unshelled before pressing (Table 2). The seven samples were sealed and stored in 120 mL brown glass bottles at room temperature.

### 3.2. Analytical Method

**Figure 8:**
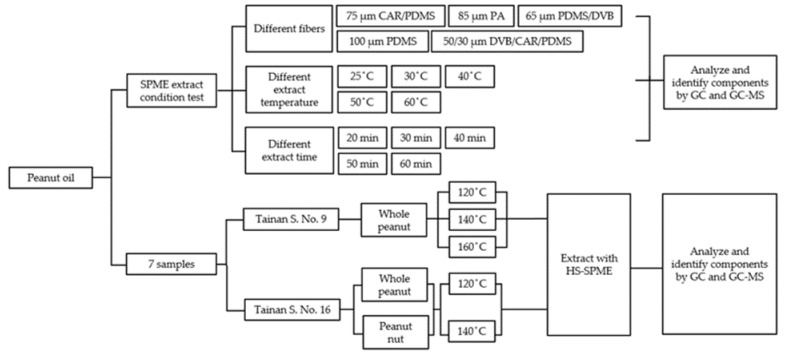
Flow diagrams of experiments performed to extract volatiles in peanut oils.

The instrument conditions and experimental methods were based on the method of Yeh et al. [33]. The experimental process is shown in Figure 8.

#### 3.2.1. SPME Extract Condition Test

(1) Testing of different fibers

The sample preparation steps included roasting the Tainan S. No. 16 peanut oil without shell and pressing at 120 °C, followed by weighing 5 g of homogenized sample into a 15 mL cylindrical glass bottle (Hole Cap PTFE/Silicone Septa) that was subsequently sealed with parafilm. Then, the HS-SPME fiber was poured into the bottle for extraction

The extraction temperature was 50 °C, and the extraction time was 40 min. After the extraction, the HS-SPME fiber was injected into the gas chromatograph and gas chromatograph–mass spectrometer for analysis. We then compared the data and selected the most appropriate absorbent fiber.

The adsorption fibers used in this experiment were all obtained from Supelco (Bellefonte, PA, USA), and the extraction fibers included

- 50/30 μm DVB/CAR/PDMS
- 65 μm PDMS/DVB
- 75 μm CAR/PDMS
- 85 μm PA
- 100 μm PDMS.

(2) Testing of different extract temperatures

The abovementioned optimal adsorption fiber was used to compare the extraction temperatures. The extraction temperatures were 25, 30, 40, 50, and 60 °C. The sample preparation is described in Section 1. The glass bottle was extracted in a water bath heater at different temperatures. The abovementioned instrument analysis was used to select the most appropriate extraction temperature.

(3) Testing of different extract times

The abovementioned optimal extraction fiber and optimal extraction temperature were both used to compare the extraction times. The extraction times were 10, 20, 30, 40, 50, and 60 min for testing. The sample preparation steps are described in Section 1. The HS-SPME extraction and the abovementioned instrument analysis were used to select the most appropriate extraction time.

#### 3.2.2. Determination of the Volatile Components in Peanut Oil 

For the determination of the volatile components, peanut oils prepared at different roasting temperatures (120 °C, 140 °C, and 160 °C) were used. Tainan S. No. 9 peanut oil was pressed into the shell, and No. 16 peanut oil was divided into shelled and unshelled peanut oil. The above seven samples (5 g) were placed into 15 mL cylindrical glass bottles and analyzed using the aforementioned HS-SPME extraction method.

### 3.3. Instrument Conditions

#### 3.3.1. Gas Chromatography (GC)

The instrument used was an Agilent (CA, U.S.A.) Model 7890 GC with an Agilent DB-1 (60 m × 0.25 mm i.d.) separation column. The injection mode was splitless. The GC heating conditions were as follows: the initial temperature was 40 °C and maintained for 1 min, raised to 150 °C at 5 °C/min and maintained for 1 min, and then raised to 200 °C at 10 °C/min and maintained for 11 min. The inlet temperature was 250 °C, the detector temperature was 300 °C, and a flame ionization detector (flame ionization detector, FID) was used for detection. The carrier was gaseous nitrogen with a flow rate of 1 mL/min.

#### 3.3.2. Gas Chromatography–Mass Spectrometry (GC-MS)

The instrument used was a Model 5977A quadrupole mass spectrometer (Mass Selective Detector, MSD) manufactured by Agilent (Palo Alto, CA, USA). The GC was Agilent Model 7890 B, and its operating conditions and the column used were the same as those of the aforementioned GC. The carrier gas was helium, the ion source temperature of the MSD was 230 °C, and the quadrupole temperature was 150 °C. The mass spectra data were compared and assessed using the Wiley 7N mass spectrum library.

#### 3.3.3. Retention Index (RI) Comparison

The GC retention indices of the volatile components in this experiment were based on a mixture of C_5_–C_25_ *n*-alkane standards (Merck, Darmstadt, Germany), and the GC retention time was used as a reference under the same conditions. They were calculated according to the method described by Kovát [34].

## 4. Conclusions

The unique flavor of peanut oil has been well received by consumers. This research developed a fast, simple, and solventless analysis method with HS-SPME and studied the differences in processing of commercial peanut oil. A total of 66 volatile components were identified in peanut oil samples. The major compound was pyrazine. When the roasting temperature was increased to 140 °C, the content of aldehydes in the peanut oil increased. Many fatty-acid oxidation products rich in furfural and 5-methylfurfural emerged when the roasting temperature increased. The primary compounds of hekei oil were pyrazines, and their content increased with an increase in roasting temperature. Additionally, heren oil was easier to oxidize. This article is the first to explore the influence of different pressing conditions on the aroma of peanut oil.

## Figures and Tables

**Table 1:** Comparisons of compounds obtained in response to different roasting temperatures for Tainan S. No. 16 and Tainan S. No. 9.

Compound ^a^	RI ^b^	Relative Content (%) ^c^						
		No.9-120 ^d^	No.9-140 ^d^	No.9-160 ^d^	Hekei-120 ^d^	Hekei-140 ^d^	Heren-120 ^d^	Heren-140 ^d^
*Aldehydes*								
Hexanal	775	6.64 ± 1.17	1.74 ± 0.04	2.21 ± 0.28	3.33 ± 0.15	4.38 ± 0.35	2.36 ± 0.21	2.01 ± 0.23
Furfural	798		10.94 ± 0.23					
(*E*)-2-Heptenal	924	3.52 ± 0.45		2.01 ± 0.15			1.89 ± 0.02	
Benzaldehyde	927				2.67 ± 0.14	4.14 ± 0.15		1.82 ± 0.18
Benzeneacetaldehyde	999		0.50 ± 0.02					
1-Ethyl-1*H*-pyrrole-2-carboxaldehyde	1009		0.31 ± 0.03					
2-Octenal	1023	1.27 ± 0.08	0.51 ± 0.01	0.74 ± 0.04	0.90 ± 0.02	1.05 ± 0.01	0.63 ± 0.01	
Nonanal	1073	4.77 ± 0.27		3.61 ± 0.13	3.85 ± 0.26	2.60 ± 0.07		3.88 ± 0.29
(*E*)-2-Nonenal	1127			2.09 ± 0.05				
4-Oxononanal	1198	0.26 ± 0.02				0.29 ± 0.06	0.22 ± 0.11	
(*E*)-2-Decenal	1235	0.43 ± 0.01						
A-Ethylidenbenzeneacetaldehyde	1237		0.79 ± 0.03	1.30 ± 0.18	0.80 ± 0.05		0.79 ± 0.05	0.75 ± 0.04
(2*E*,4*Z*)-Decadienal	1265			0.40 ± 0.07			0.27 ± 0.00	0.27 ± 0.02
(*2E*,4*E*)-Decadienal	1285	0.25 ± 0.01	0.36 ± 0.01	0.52 ± 0.38	0.60 ± 0.03	0.37 ± 0.01	0.39 ± 0.01	0.50 ± 0.02
Vanillin	1351	0.17 ± 0.04	0.14 ± 0.00		0.14 ± 0.03			
*Alcohols*								
2-Furanmethanol	827		3.92 ± 0.02	1.49 ± 0.15		1.81 ± 0.05		
1-Octanol	1042					0.47 ± 0.06		
Benzene ethanol	1076					0.53 ± 0.05		
Furans								
Acetylfuran	876	0.80 ± 0.13	0.50 ± 0.01	0.69 ± 0.04	0.84 ± 0.04	0.59 ± 0.02	0.46 ± 0.03	0.62 ± 0.14
2-Methyl-5-Formylfuran	926		5.17 ± 0.03					
*Hydrocarbons*								
Ethenylbenzene	870	0.48 ± 0.07						
Undecane	1100		0.74 ± 0.02	1.16 ± 0.05	1.32 ± 0.11	1.01 ± 0.04	1.01 ± 0.04	1.07 ± 0.05
Naphthalene	1162					0.27 ± 0.01		0.44 ± 0.14
Dodecane	1200		0.78 ± 0.02	1.27 ± 0.02	1.48 ± 0.12	1.02 ± 0.01	1.16 ± 0.05	1.14 ± 0.03
Tridecane	1300		0.55 ± 0.03	0.95 ± 0.15	1.03 ± 0.09		0.72 ± 0.06	0.77 ± 0.05
3-Phenylthiophene	1384		0.18 ± 0.01					
Tetradecane	1400			0.06 ± 0.01	0.41 ± 0.03	0.23 ± 0.06	0.25 ± 0.05	0.31 ± 0.08
Butylated hydroxytoluene	1488	0.74 ± 0.17	0.34 ± 0.11	0.45 ± 0.17	1.12 ± 0.05		0.50 ± 0.08	0.86 ± 0.18
*Ketones*								
Methyl pyrrol-2-yl ketone	1015	2.32 ± 1.62	1.46 ± 0.10	1.90 ± 0.05	1.26 ± 0.02	1.45 ± 0.07	1.65 ± 0.08	1.48 ± 0.11
Acetophenone	1027	0.61 ± 0.09	0.64 ± 0.01	0.74 ± 0.01	0.59 ± 0.11	0.68 ± 0.01	0.37 ± 0.01	
γ-Nonalactone	1313	0.25 ± 0.01					0.15 ± 0.00	
*Phenols*								
Phenol	949		0.48 ± 0.01					
*p*-Cresol	1041			0.31 ± 0.05				
*p*-Ethylphenol	1133	12.85 ± 0.60	0.39 ± 0.01	2.30 ± 0.25	7.21 ± 0.14	0.26 ± 0.01	1.67 ± 0.12	11.36 ± 0.44
*p*-Vinylphenol	1183		11.80 ± 0.32	10.00 ± 1.32	2.54 ± 0.05	6.53 ± 0.56	6.73 ± 0.46	2.81 ± 0.22
4-Ethyl-2-methoxyphenol	1252	2.78 ± 0.07			0.84 ± 0.03		0.33 ± 0.01	
Sesamol	1278			0.23 ± 0.13				
2-Methoxy-4-vinylphenol	1281	0.30 ± 0.02	2.82 ± 0.07	2.67 ± 0.33	1.30 ± 0.05	1.59 ± 0.06	1.63 ± 0.14	1.32 ± 0.07
*p*-(1-Methylpropyl)phenol	1287			0.39 ± 0.06	0.36 ± 0.02	0.14 ± 0.01		
*Pyrazines*								
Pyrazine	725			1.47 ± 0.16				
2-Methylpyrazine	796	3.78 ± 0.48		5.19 ± 0.33	3.52 ± 0.12	5.60 ± 0.26	5.68 ± 0.27	3.69 ± 0.19
2,5-Dimethylpyrazine	881	9.48 ± 1.26	11.43 ± 0.15	12.98 ± 1.34	10.80 ± 0.82	14.65 ± 0.67	20.14 ± 0.58	13.54 ± 1.57
Vinylpyrazine	897		0.16 ± 0.00					
2-Ethyl-6-methylpyrazine	966	3.49 ± 0.15	4.96 ± 0.06	3.42 ± 0.23	3.96 ± 0.72	3.81 ± 0.17	2.86 ± 0.04	3.46 ± 0.05
2-Ethyl-3-methylpyrazine	970	9.63 ± 0.92	6.20 ± 0.10	8.25 ± 1.04		8.66 ± 0.29	11.60 ± 0.29	10.96 ± 0.80
2-Methyl-6-vinylpyrazine	981		2.51 ± 0.01	0.85 ± 0.06		2.56 ± 0.07	0.81 ± 0.01	1.12 ± 1.18
2-Methyl-5-vinylpyrazine	985			2.14 ± 0.02				4.34 ± 2.18
2-Ethyl-3,6-dimethylpyrazine	1048	4.01 ± 0.23	3.54 ± 0.02		5.26 ± 0.36			4.43 ± 0.47
2-Ethyl-3,5-dimethylpyrazine	1054		1.75 ± 0.03	7.35 ± 0.60		7.41 ± 0.23	6.75 ± 0.19	1.69 ± 0.13
5-Ethyl-2,3-dimethylpyrazine	1055						2.04 ± 0.06	
2,5-Diethylpyrazine	1058	0.32 ± 0.01	0.31 ± 0.00	0.39 ± 0.01	0.35 ± 0.01	0.39 ± 0.01	0.42 ± 0.01	0.37 ± 0.02
6,7-Dihydro-5*H*-cyclopentapyrazine	1066		1.24 ± 0.01					
Dimethyl-2-vinylpyrazine	1067	1.18 ± 0.03		1.47 ± 0.06		1.37 ± 0.01		
6-Methyl-2-acetylpyrazine	1080		1.02 ± 0.04	0.91 ± 0.05	0.33 ± 0.00	0.79 ± 0.04	0.49 ± 0.11	0.43 ± 0.02
5*H*-5-Methyl-6,7-dihydrocyclopentapyrazine	1102		0.56 ± 0.00		0.44 ± 0.01	0.73 ± 0.01	0.53 ± 0.01	0.55 ± 0.04
2-Methyl-3,5-diethylpyrazine	1128						1.69 ± 0.08	1.51 ± 0.07
3,5-Dimethyl-2-isobutylpyrazine	1176		0.20 ± 0.01					
2,5-Dimethyl-3-isobutylpyrazine	1178					0.20 ± 0.02	0.39 ± 0.01	
3,5-Dimethyl-6,7-dihydro-5*H*-cyclopentapyrazine	1189					0.37 ± 0.01		
2,5-Dimethyl-3-isopentylpyrazine	1295					0.15 ± 0.01		
*Pyridines*								
β-Methoxypyridin	961		0.21 ± 0.01	0.73 ± 0.12				0.92 ± 0.02
2-Methyl 5*H*-6,7-dihydrocyclopentapyrazine	1158			0.70 ± 0.04	0.48 ± 0.03	0.67 ± 0.00		0.20 ± 0.17
2-Propylpyridine	1171			0.54 ± 0.07	0.27 ± 0.01			
*Others*								
Benzeneacetonitrile	1085				0.36 ± 0.00		0.43 ± 0.01	0.33 ± 0.03
Trigonelline	1096					0.32 ± 0.02		
Methyl nicotinate	1095		0.46 ± 0.01	0.45 ± 0.04			0.28 ± 0.02	0.31 ± 0.04

^a^ Volatile components were identified on the basis of gas chromatographic retention indices, mass spectra from Wiley MS Chemstation Libraries (6th edition, G1034, Rev. C.00.00, Hewlett–Packard); ^b^ Retention indices using *n*-paraffin (C_5_–C_25_) as references; ^c^ values are the mean ± SD of triplicates; ^d^ different samples of peanut oils.

**Table 2:** The study of collections of taxa currently assigned to peanut oil.

Sample Name	Variety	Pressure Oil Part	Roasting Temperature (°C)
**No.9-120**	Tainan Selected No. 9	Shell and nut	120
**No.9-140**	Tainan Selected No. 9	Shell and nut	140
**No.9-160**	Tainan Selected No. 9	Shell and nut	160
**Hekei-120**	Tainan Selected No. 16 (Hei King Kong)	Shell and nut	120
**Hekei-140**	Tainan Selected No. 16 (Hei King Kong)	Shell and nut	140
**Heren-120**	Tainan Selected No. 16 (Hei King Kong)	nut	120
**Heren-140**	Tainan Selected No. 16 (Hei King Kong)	nut	140

## Data Availability

Not applicable.

## Sample Availability

Samples of the compounds are not available from the authors.
